# Evaluating the Effect of Repetitive Transcranial Magnetic Stimulation on Disorders of Consciousness by Using TMS-EEG

**DOI:** 10.3389/fnins.2016.00473

**Published:** 2016-10-20

**Authors:** Yang Bai, Xiaoyu Xia, Jiannan Kang, Xiaoxiao Yin, Yi Yang, Jianghong He, Xiaoli Li

**Affiliations:** ^1^Department of Automation, Institute of Electrical Engineering, Yanshan UniversityQinhuangdao, China; ^2^Department of Neurosurgery, PLA Army General HospitalBeijing, China; ^3^Department of Biomedical Engineering, Medical School, Tsinghua UniversityBeijing, China; ^4^Department of Biomedical Engineering, Institute of Electronic Information Engineering, Hebei UniversityBaoding, China; ^5^State Key Laboratory of Cognitive Neuroscience and Learning & IDG/McGovern Institute for Brain Research, Beijing Normal UniversityBeijing, China; ^6^Center for Collaboration and Innovation in Brain and Learning Sciences, Beijing Normal UniversityBeijing, China

**Keywords:** TMS-EEG, disorder of consciousness, rTMS, perturbation complexity index, EEG

## Abstract

**Background:** The modulation efficacy of Transcranial magnetic stimulation (TMS) on consciousness improvement of patient with disorder of consciousness (DOC) has not been definitely confirmed.

**Objective:** This study proposes TMS-EEG to assess effects of repetitive TMS (rTMS) on brain modulation of DOC.

**Methods:** Twenty sessions of 10 Hz rTMS were applied over the dorsolateral prefrontal cortex for a patient with DOC. Measures of Coma Recovery Scale-Revised (CRS-R) score, TMS-evoked potential (TEP), perturbation complexity index (PCI), and global mean field power (GMFP) were used to evaluate the consciousness level of the patient at three intervals: before the rTMS protocol (T0), immediately after one session rTMS (T1), and immediately after 20 sessions (T2).

**Results:** It was found that the patient was diagnosed of a minimally conscious state minus (MCS-) by means of CRS-R at the interval of T0, however the TEP and PCI indicated the patient was vegetative state (VS). At the interval of T1, there was not any clinical behavioral improvement in CRS-R, but we could find significant changes in TEP, PCI, and GMFP. At the interval of T2 there was a significant increase of consciousness level according by CRS-R score, PCI value, TEP, and GMFP after 20 sessions of 10 Hz rTMS on the patient with DOC.

**Conclusions:** We demonstrated that TMS-EEG might be an efficient assessment tool for evaluating rTMS protocol therapeutic efficiency in DOC.

## Introduction

Although, some studies have attempted to demonstrate some pharmacologic or nonpharmacologic effects, until now there were no evidence-based guidelines regarding the treatment of patients with DOC (Bernat et al., 2006). Recently, few case reports have addressed the application of rTMS on consciousness improve in patients of vegetative state (VS) or minimally consciousness state (MCS) (Lefaucheur et al., 2014). Effects of high frequency rTMS on MCS have been reported in several patients. A recent case report demonstrated that a patient in MCS has meaningful behaviors increase after received three sessions 20 Hz rTMS on the primary motor cortex (M1), it suggested that the rTMS might improve consciousness of MCS patient (Piccione et al., 2011). In a previous study (Manganotti et al., 2013), six patients in VS and MCS received one session of 20 Hz rTMS on M1 region, and one MCS patient has significant clinical and EEG modification. In a study (Naro et al., 2015), by using a protocol of 10 Hz rTMS over dorsolateral prefrontal cortex (DLPFC), three of ten patients with MCS have significant clinical improvement. Also, it was found that there was a significant improvement for VS patients after rTMS modulation. Until now, a case study was only reported that, when TMS deliver to DLPFC, a 30 sessions rTMS protocol could improve neurobehavioral change of VS patient (Louise-Bender Pape et al., 2009). Basically, rTMS modulation for DOC patient might be available.

How to evaluate the modulation of rTMS on the DOC patient is a critical issue. The current gold standard for assessing consciousness state is based on the standard behavioral assessment (Monti and Sannita, 2016). However, possible confounding factors and mechanisms underlying impaired brain function may not be fully considered. The absence of behavioral evidence of command following was not necessarily indicative of the true absence of consciousness (Owen et al., 2006). It was reported that the behavioral abilities could fluctuate across time which would cause mis-diagnostic (Monti and Sannita, 2016). Recently, several “stimulate-response” methods, such as short-latency evoked potentials (Cavinato et al., 2009; Ragazzoni et al., 2013), event-related potentials (Kotchoubey et al., 2005; Rohaut et al., 2015), and motor evoked amplitude (Naro et al., 2015) have been used as more objective assessment methods for the consciousness level of patient with DOC. However, the proposed methods did not consider the integrity of sensory or peripheral nerve pathways of DOC. Thus, it is necessary to develop a new and reliable method to accurately assess the clinical variety in DOC treatment.

Recently TMS-EEG was proposed to evaluate the consciousness state in severely brain-injured and disable of communication patients (Rosanova et al., 2012), and it could invariably trigger complex activations that sequentially involved distant cortical areas ipsi- and contralateral to the site of stimulation in MCS which was different from VS. TMS-EEG technique could directly detect the relationship between non-invasive stimulation and cortical response, and it should not depend on the condition of participant.

Although, TMS-EEG has been demonstrated helpful in differentiating MCS from VS, there were rarely studies using this technology to assess the efficiency of present therapy in DOC. The primary aim of this study was to explore TMS-EEG evidence in consciousness recovery during a therapy of rTMS protocol. The second aim was to support an example of using TMS-EEG to assess the therapy efficiency in DOC.

## Background

The patient is a woman age 47 with brain injury induced by hypertensive cerebral hemorrhage of right basal ganglia region. Post-injury, the patient remained behaviorally unresponsive for a period of 2 months based on available records and appeared unstable source location of pain after 8 weeks of injury. At about 10 weeks post-injury, the patient was transferred to a comprehensive inpatient brain injury rehabilitation program where physical, occupational, speech, and related therapies were performed for the next 7 months. But no distinct behaviorally improvement during this period. It was 9 months after-injury when she began to accept rTMS therapy, she was diagnosed as MCS- by CRS-R. She could open her eyes spontaneously, and blink when received big sound stimulation like clap but can't locate the sound source, noxious stimulation withdrawing the respective limb from the pain source. She can't sound and had no any commands following response, her mouth had reflex movement. Her caregivers reports that, she had relative stable sleeping time in afternoon and after midnight.

## Materials and methods

### Subjects

Written informed consent to participate in the study was obtained from the patient's caregivers. In order to indicate the difference of TMS-evoked potential (TEP) between the patient with DOC and controls, five age matched female healthy subjects age 43–50 participated to this study to obtain the mean control TEP. Written informed consent to subjects in the study was obtained. The present study was approved by ethics committee of Beijing Army General Hospital.

### Stimulation protocol

**Figure 1:**
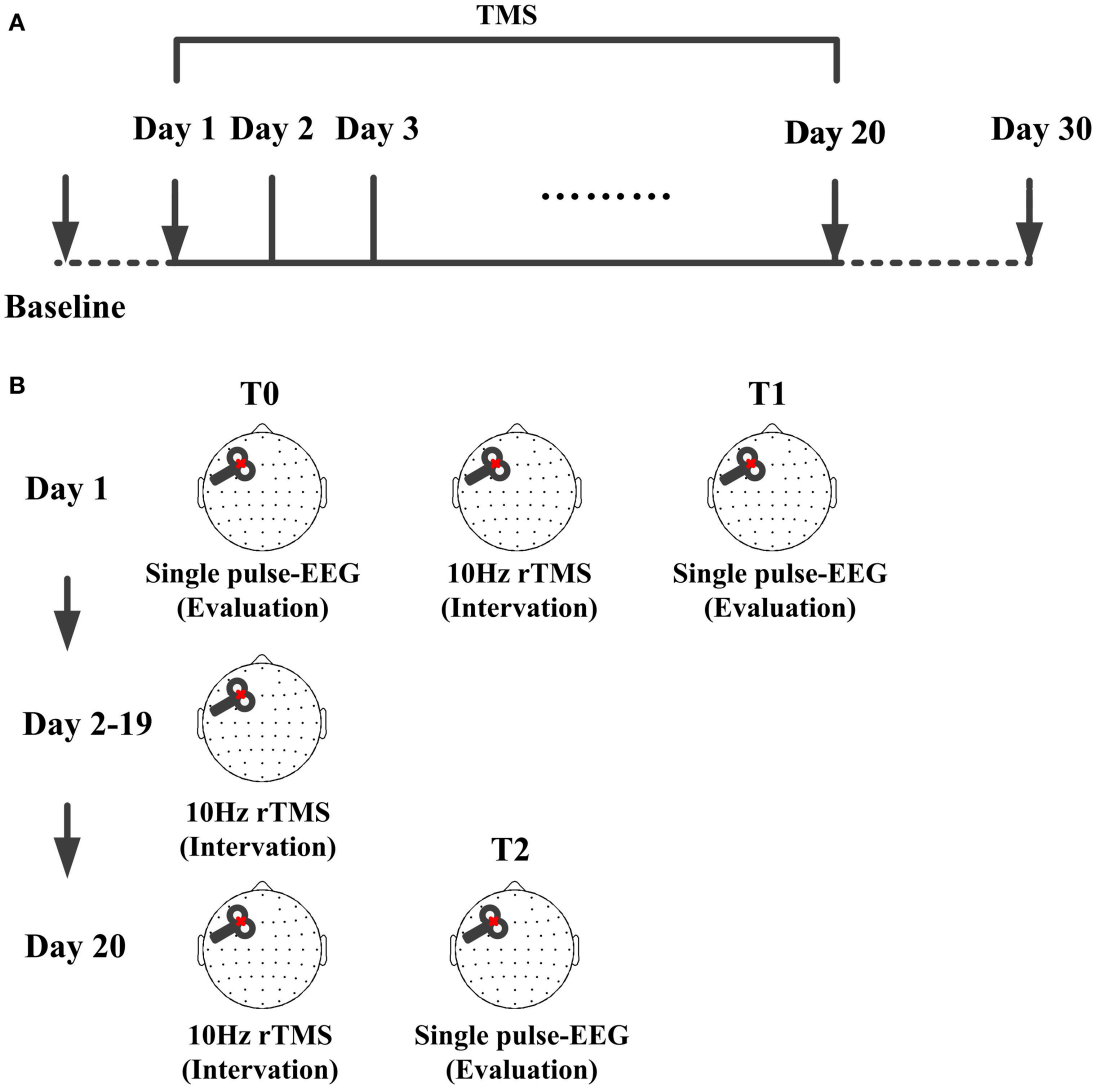
**TMS protocol for the patient. (A)** Time points of the protocol, TMS were delivered at left-DLPFC lasting for 20 consecutive days. CRS-R score was assessed each day from the baseline to day 30. **(B)** Single pulse TMS evoked EEG recording before the protocol were used as baseline (T0) and that immediately after one session were used as the one session assessment (T1). From day 2 to day 19, there were no TMS and EEG evaluation. Single pulse TMS evoked EEG recorded immediately after the 20 sessions were used as assessment of the whole protocol (T2).

The process of the intervention and evaluation could be found in Figure 1. The patient received active 10 Hz rTMS over left DLPFC. Different from similar study (Naro et al., 2015), our protocol lasted 20 consecutive days. Daily sessions of intervention consisted of 1000 pulses (10 Hz) at an intensity of 90% RMT. The stimulation of one session included 10 trains, each train lasted 10 s with a 60 s inter-train pause. The rise time of the magnetic monophasic stimulus was about 100 μs and time to zero was about 800 μs. TMS pulses were delivered using a Magstim *R*^2^ stimulator with a 70 mm figure-of-eight coil (Magstim Company Limited, Whitland, UK). Stimulation intensity varied across this experiment was determined relative to the resting motor threshold (RMT), defined as the lowest TMS intensity which could evoke at least 5 out of 10 EMG with an amplitude >50 μV peak-to-peak in the relaxed first dorsal interosseous muscle of the right hand. To avoid contamination of TMS-evoked potential by auditory potentials evoked by the click associated with the TMS discharge, patient was wearing inserted earplugs continuously playing a masking noise.

### CSR-R

The CRS-R is a tool to characterize the level of consciousness and to monitor neuro-behavioral recovery in patients with DOC (Giacino et al., 2004). Scoring is based on the presence or absence of specific behavioral responses to sensory stimuli administered in a standardized manner, with low item represents reflexive activity and high items represent cognitively mediated behaviors. In this study, CRS-R was assessed daily from 1 day before rTMS protocol to30 days after this protocol.

### TMS-EEG recordings

In the experiment, we used TMS-compatible EEG recorder (BrainAmp 64 MRplus, BrainProducts), EEG was continuously acquired from 62 channels with positions of the international 10–10 system. The equipment used TMS-compatible sintered Ag/AgCl-pin electrodes. We set a band-pass filtered at DC to 1000 Hz in the recorder, and the EEG signal was digitized at a sampling rate of 2.5 kHz. During the experiment, the skin/electrode impedance was maintained below 5 kΩ. EEG was recorded in day 1 and day 20. As shown in Figure 1B, at T0, 200 single pulses were delivered before the protocol as baseline assessment and TMS evoked EEG immediately recorded after 10 Hz rTMS to evaluate the efficacy of one session rTMS. At T2, 200 single pulses were delivered immediately after rTMS to assess the performance of whole protocol. EEG recordings were carried out while patients were behaviorally awake (eyes open, EO) during the modulation and assessment. If the patient showed signs of sleepiness (prolonged eye closure, EC), the CRS-R arousal facilitation protocol was applied, or the experiment was suspended.

### EEG analysis

#### Evoked potential by TMS

Off-line analysis was performed with EEGLAB 12.0.2.5b, running in a MATLAB environment (Version 2013b, MathWorks Inc., Natick, USA). The continuous EEG signal was segmented into epochs starting the TMS pulse onset and ending 300 ms (Massimini et al., 2005; Ferrarelli et al., 2010; Ferreri et al., 2011) after it. After this, data 20 ms after TMS pulse were removed from each trial to exclude the TMS artifact through the cubic interpolation function of MATLAB (Thut et al., 2011). Independent component analysis (ICA) function was used to identify the TMS unrelated artifacts (such as eye movement and muscle artifacts). Then each component was visually inspected in terms of scalp distribution, frequency, timing, and amplitude. The components deemed as artifact were removed with ICA (Casula et al., 2014). The 50 Hz artifact was removed from remaining trials using a notch filter. Then, EEG data were average referenced; down-sampled to 500 Hz, band pass filtered (1–80 Hz), and baseline corrected over 300 ms pre-stimulus. Single trails were carefully inspected to ensure absence of residual TMS artifacts. Each TMS-evoked response was obtained by averaging 150–200 artifact-free trials.

#### Perturbation complexity index

An index of consciousness, called the perturbation complexity index (PCI), was applied to evaluate the consciousness level of the patient. The PCI was proposed before (Casali et al., 2013), the calculation mainly includes three steps. Firstly, TMS evoked cortical perturbation (300 ms after TMS pulse) which was recorded by high-density EEG (62 channels in this study). Then source modeling was performed and nonparametric statistics extracted a binary matrix [*SS*(*x, t*)] which describes the spatiotemporal pattern of activation caused by the TMS perturbation. At last, the Lempel-Ziv complexity index was used to compress the matrix. The PCI index was calculated as the normalized Lempel-Ziv complexity. The PCI is expected to be low if there is reduced interaction among cortical areas and will be high if interaction of the cortical areas increased. As suggested (Casali et al., 2013), the PCI values in VS were range of 0.19–0.31 and in MCS were range of 0.32–0.49.

#### Global mean field power

In order to obtain the overall amount of electrical activity induced by TMS, the global mean field power (GMFP) was calculated with the multichannel average signals as follows (Lehmann and Skrandies, 1980):

(1) $$\begin{array}{r} \text{GMFP}\left(\text{t}\right)=\sqrt{\frac{\sum_{i}^{k}{\left(V_{i}\left(t\right)-V_{mean}\left(t\right)\right)}^{2}}{k}} \end{array}$$

where *k* means the number of channels, *V*_*i*_ means the amplitude of channel I, and *V*_*mean*_ is the mean value of the amplitude across all channels.

## Results

### CRS-R

CRS-R were used to assess the consciousness level of the patient (Table 1). CRS-R score of 8 was marked at baseline. With the rTMS protocol starting, the score remained unchanged for the first 8 days. Although the patient care claimed that the patient showed more excitation, more sensitive to stimulation and less sleeping time, there were no significant clinical behavioral improvement expressed in CRS-R. In the day 9, some simple finger movements were found, and the CRS-R score increased to 10. From the day 15 to 20, the patient represented stable simply movement following the command with score of 13, and her eyes could track movement of objects like mobile phone with video. In the last week of this protocol, there was one score increased for her concentration on something for a time. The shadow area in the Table 1 shows the CRS-R values in 20 days with 10 Hz rTMS. In the shadow area, the consciousness level of the patient arose from MCS− to MCS+ with score from 8 to 13.

**Table 1:** **Data of the CRS-R score in this protocol**.

**Time**	**CRS-R**							**CS**
**(day)**	**Auditory**	**Visual**	**Motor**	**Oro-motor**	**Comm**	**Arousal**	**Total**	
Baseline	1	1	3	1	0	2	8	MCS−
1–8	1	1	3	1	0	2	8	MCS−
9–14	3	1	3	1	0	2	10	MCS+
15–20	4	3	3	1	0	2	13	MCS+
20–22	4	3	3	1	0	2	13	MCS+
23–30	4	3	3	1	0	3	14	MCS+

Shadow area shows the score during rTMS sessions. Comm, communication; CS, consciousness state.

### TMS-EEG

#### TEP

**Figure 2:**
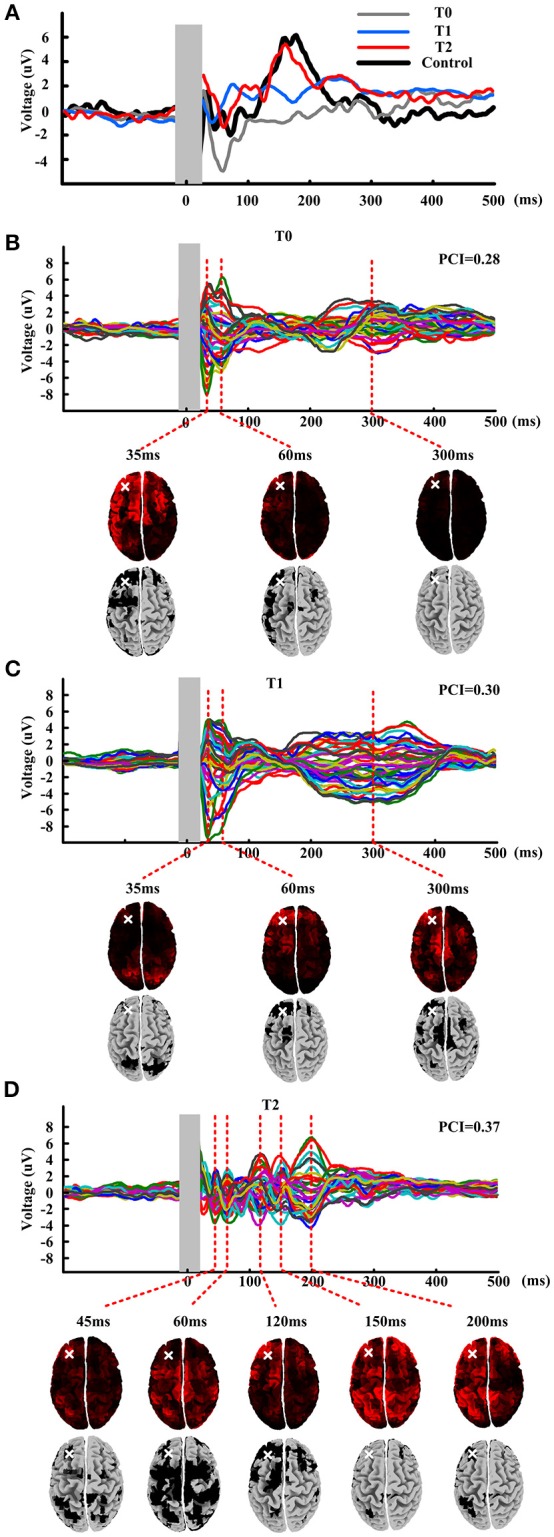
**TMS evoked potential and butterfly plots at three time points: T0, T1, and T2. (A)** TMS evoked potentials of mean of healthy subjects and patient at three time points. Shadow area means 10 ms before to 20 ms after the TMS onset. **(B–D)** Butterfly plots of patient at three time points. Source modeling corresponding to each TEP peaks is given under the butterfly plots. Last row of each figure gives the significant activation distribution. White cross shows the stimulation site.

Single pulse evoked potential over DLPFC was calculated at T0, T1, and T2, could be seen in Figure 2A. Black line shows the TEP over DLPFC calculating of mean of healthy subjects. We could see that the TEP of T0 shows simple positive-negative EEG response with positive peak at about 35 ms and negative peak at about 60 ms. The TEP of the T1 shows more channels' response activity than T0 while the TEP of the T2 shows more complex than the T1 with bigger amplitude of the fluctuation. Comparing with the TEP of the healthy subjects, the TEP of T2 appears similar fluctuation: positive peak at about 180 ms, which never occurred before.

Figures 2B–D show the butterfly plots of the TEP at three time point, respectively. There have nearly same temporal distribution of the peaks for T0 and T1, but very different from T2. Then PCI was calculated for quantifying the TEP. The first row under the butterfly plot shows the source modeling of corresponding TEP peaks. And then nonparametric statistics was performed to obtain a significant activation distribution (last row of each figure), where the black regions in the cortical represent significant cortical activation induced by the stimulation. The activation distribution of T0-T1-T2 indicate a trend of from local to global and from ipsilateral to contralateral. After compressing the binary matrix, the PCI was obtained. At T0, the PCI value was 0.28 and after one session rTMS, the PCI value raised to 0.30. After all the protocol, the PCI value raised to 0.37.

#### GMFP

**Figure 3:**
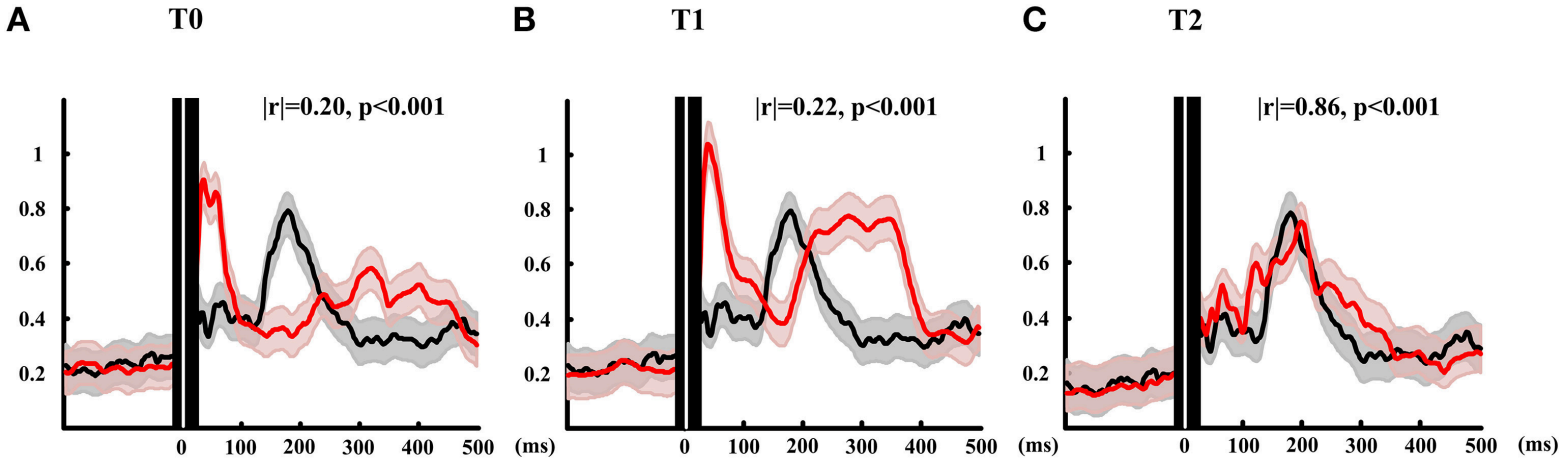
**GMFP calculated of patient at three time points and the mean of healthy subjects**. Red lines show the GMFP curves of the patient at T0 **(A)**, T1 **(B)**, and T2 **(C)**. Black lines show the mean GMFP curves of healthy subjects. The gray and red shadow means standard deviation of patient and healthy subjects, respectively. Black area with white line show 10 ms before and 20 ms after TMS onset.

The GMFP is depicted in Figure 3. The black line shows the mean GMFP of the healthy subjects and the red lines show the GMFP of the patient at three different time. The correlation coefficient (Matlab code: corrcoef.m) were calculated of the GMFP after stimulation between the patient and the healthy subjects. At T0, the correlation is 0.2 and T1 the correlation arose to 0.22. Different from the mean value of healthy subjects, the activation power of the global brain for the T0 and T1 mainly distributed within 100 ms after stimulation. And during the time window from 250 to 400 ms, the global brain power were activated at T0 and T1 while the healthy subject didn't show any activation. After all the rTMS protocol, the GMFP pattern of the patient was similar with the mean GMFP of healthy subjects with correlation 0.86. Meanwhile, the time window of main activation power of the patient was well-matched with the healthy subjects, and main power occurred within 300 ms after stimulation.

## Discussion

### TEP

Measuring the EEG responses to TMS to differentiate different consciousness states had been proposed (Rosanova et al., 2012). Similar with that observed in unconscious sleeping or anesthetized subjects (Massimini et al., 2005; Ferrarelli et al., 2010), in awake VS patients, TMS triggered a simple, local slow response that indicated a breakdown in effective connectivity. While TMS triggered complex activations that sequentially involved distant cortical areas ipsilateral and contralateral stimulation site in MCS patients. The baseline TEP of the patient in this study showed a relative simple and slow curve similar with VS introduced by Rosanova et al. (2012). It did exactly matched the clinical behavioral result which was diagnosed as MCS- with CRS-R score of 8. The possible reason is due to the fluctuations in behavioral abilities across time. After one session rTMS, TMS triggered more channels' activity. After all the rTMS protocol, the TEP represented a more complex curve, which combined with the theory linking integration and differentiation to consciousness indicated a positive modulation effects of rTMS on this patient.

### PCI

The PCI is a measurement to quantify the complexity of TEP and it measures the amount of information integration and differentiation within the brain's response to perturbation. The PCI value of the patient in this study arose from 0.28 to 0.37. As described by Casali et al. (2013) the PCI ranged from 0.19 to 0.31 for a stable clinical diagnosis of VS, and ranged from 0.32 to 0.49 for MCS patients. Therefore, according to the PCI values, the patient was diagnosed as a VS before rolling in this TMS protocol, and it was still staying in VS after one rTMS session despite an increase of PCI value. After all the protocol rTMS, the PCI indicated that the patient was staying in MCS+. The TEP results and CRS-R score were consistent with the variation tendency of PCI and both indexes indicated an improvement of consciousness state. But the PCI values showed VS state of the patient at baseline which was not agreement with the CRS-R score. Consistent with the TEP results, the PCI values also showed that patient was still in vegetate state, although appeared much fast oscillation in TEP after one session rTMS, it indicated the patient didn't emerge from vegetate state. Indeed, the boundary of PCI used to differentiate VS from MCS was not perfectly accurate as the study just enrolled few patients for calculating, six for VS and six for MCS. But the PCI values might be useful as a significant potential diagnostic tool for consciousness evaluation.

### GMFP

The GMFP results of this study showed that, at a global level, one session rTMS over the left DLPFC increased cortical excitability in temporal windows of 30–100 and 200–400 ms after stimulation. Interestingly, when comparing with healthy subjects, there was a global activation after 300 ms of stimulation for baseline and one session TEP, which nearly impossibly occurred in healthy subjects even in consciousness reduced states such as anesthesia (Ferrarelli et al., 2010) and sleep state (Massimini et al., 2005). The possible reason is that the “overtime” activation may be induced by abnormally electrical transmission evoked by damaged brain region. Then after 20 sessions rTMS, the brain activation pattern (amplitude and time) was tend to well-matched with the healthy brain. Hence, combined with the clinical behavioral assessment in CRS-R scores, we suggested that GMFP might be also a significant marker for consciousness recovery of DOC.

Overall, although there had some diversity in evaluating the base line and one session, all the assessment methods proposed in this case study consistently indicated that the consciousness state was improved after all the rTMS protocol. This divergence of the baseline assessment might be induced by fluctuation of state of the patient and the sensitivity of assessment method should be tested in quantity application. On the other hand, as demonstrated in Naro et al. (2015), we suggest that one session rTMS indeed has transiently improvement but it may difficult leading to permanent clinical behavioral change. Thereby, in this study, we used 20 sessions to use the accumulation efficacy of the rTMS modulation. The incubation time was 8 days in this study but 10 days was reported in a patient in Louise-Bender Pape et al. (2009), we think that this modification efficiency may be variable in individual level and depend on the time and intensity of rTMS.

## Concluding remarks

This was first study on reporting TMS-EEG based characteristic of consciousness recovery during rTMS protocol. The results indicated that the TMS-EEG might lead to more objectively evaluation of consciousness and might be an efficient assessment tool for rTMS protocol therapeutic efficiency evaluation. Our study was an example of using TMS-EEG method to assess an therapy efficiency in DOC. And we suggest that methods of TMS-EEG supported in this study may facilitate therapy improvement in DOC.

## Author contributions

All authors listed, have made substantial, direct and intellectual contribution to the work, and approved it for publication. YB had full access to all the data in the study and takes responsibility for the integrity of the data and the accuracy of the data analysis. Study concept and design, acquisition, analysis, or interpretation of data: YB and XX. Administrative, technical, or material support: XX, JK, XY, and YY. Study supervision and obtained funding: JH and XL.

## Funding

This research was supported by the National Natural Science Foundation of China (No. 61273063, No. 81230023), Beijing Municipal Science & Technology Commission (No. Z141107002514111), Beijing Municipal Commission of Education and Innovation Cultivation Fund of the PLA Army General Hospital (No. 2015-LC-09).

### Conflict of interest statement

The authors declare that the research was conducted in the absence of any commercial or financial relationships that could be construed as a potential conflict of interest.

## References

[B1] Bernat J. L. D'Alessandro A. M. Port F. K. Bleck T. P. Heard S. O. Medina J.. (2006). Report of a National Conference on Donation after cardiac death. Am. J. Transplant. 6, 281–291. 10.1111/j.1600-6143.2005.01194.x 16426312

[B2] Casali A. G. Gosseries O. Rosanova M. Boly M. Sarasso S. Casali K. R.. (2013). A theoretically based index of consciousness independent of sensory processing and behavior. Sci. Transl. Med. 5, 198ra105. 10.1126/scitranslmed.3006294 23946194

[B3] Casula E. P. Tarantino V. Basso D. Arcara G. Marino G. Toffolo G. M.. (2014). Low-frequency rTMS inhibitory effects in the primary motor cortex: insights from TMS-evoked potentials. Neuroimage 98, 225–232. 10.1016/j.neuroimage.2014.04.065 24793831

[B4] Cavinato M. Freo U. Ori C. Zorzi M. Tonin P. Piccione F.. (2009). Post-acute P300 predicts recovery of consciousness from traumatic vegetative state. Brain Inj. 23, 973–980. 10.3109/02699050903373493 19831494

[B5] Ferrarelli F. Massimini M. Sarasso S. Casali A. Riedner B. A. Angelini G.. (2010). Breakdown in cortical effective connectivity during midazolam-induced loss of consciousness. Proc. Natl. Acad. Sci. U.S.A. 107, 2681–2686. 10.1073/pnas.0913008107 20133802 PMC2823915

[B6] Ferreri F. Pasqualetti P. Määttä S. Ponzo D. Ferrarelli F. Tononi G.. (2011). Human brain connectivity during single and paired pulse transcranial magnetic stimulation. Neuroimage 54, 90–102. 10.1016/j.neuroimage.2010.07.056 20682352

[B7] Giacino J. T. Kalmar K. Whyte J. (2004). The JFK coma recovery scale-revised: measurement characteristics and diagnostic utility. Arch. Phys. Med. Rehabil. 85, 2020–2029. 10.1016/j.apmr.2004.02.033 15605342

[B8] Kotchoubey B. Lang S. Mezger G. Schmalohr D. Schneck M. Semmler A.. (2005). Information processing in severe disorders of consciousness: vegetative state and minimally conscious state. Clin. Neurophysiol. 116, 2441–2453. 10.1016/j.clinph.2005.03.028 16002333

[B9] Lefaucheur J.-P. André-Obadia N. Antal A. Ayache S. S. Baeken C. Benninger D. H.. (2014). Evidence-based guidelines on the therapeutic use of repetitive transcranial magnetic stimulation (rTMS). Clin. Neurophysiol. 125, 2150–2206. 10.1016/j.clinph.2014.05.021 25034472

[B10] Lehmann D. Skrandies W. (1980). Reference-free identification of components of checkerboard-evoked multichannel potential fields. Electroencephalogr. Clin. Neurophysiol. 48, 609–621. 10.1016/0013-4694(80)90419-8 6155251

[B11] Louise-Bender Pape T. Rosenow J. Lewis G. Ahmed G. Walker M. Guernon A.. (2009). Repetitive transcranial magnetic stimulation-associated neurobehavioral gains during coma recovery. Brain Stimul. 2, 22–35. 10.1016/j.brs.2008.09.004 20633400

[B12] Manganotti P. Formaggio E. Storti S. F. Fiaschi A. Battistin L. Tonin P.. (2013). Effect of high-frequency repetitive transcranial magnetic stimulation on brain excitability in severely brain-injured patients in minimally conscious or vegetative state. Brain Stimul. 6, 913–921. 10.1016/j.brs.2013.06.006 23928101

[B13] Massimini M. Ferrarelli F. Huber R. Esser S. K. Singh H. Tononi G. (2005). Breakdown of cortical effective connectivity during sleep. Science 309, 2228–2232. 10.1126/science.1117256 16195466

[B14] Monti M. M. Sannita W. G. (2016). Brain Function and Responsiveness in Disorders of Consciousness. Chem: Springer International Publishing.

[B15] Naro A. Russo M. Leo A. Bramanti P. Quartarone A. Calabrò R. S. (2015). A single session of repetitive transcranial magnetic stimulation over the dorsolateral prefrontal cortex in patients with unresponsive wakefulness syndrome: preliminary results. Neurorehabil. Neural Repair 29, 603–613. 10.1177/1545968314562114 25539781

[B16] Owen A. M. Coleman M. R. Boly M. Davis M. H. Laureys S. Pickard J. D. (2006). Detecting awareness in the vegetative state. Science 313, 1402. 10.1126/science.1130197 16959998

[B17] Piccione F. Cavinato M. Manganotti P. Formaggio E. Storti S. F. Battistin L.. (2011). Behavioral and neurophysiological effects of repetitive transcranial magnetic stimulation on the minimally conscious state: a case study. Neurorehabil. Neural Repair 25, 98–102. 10.1177/1545968310369802 20647501

[B18] Ragazzoni A. Pirulli C. Veniero D. Feurra M. Cincotta M. Giovannelli F.. (2013). Vegetative versus minimally conscious states: a study using TMS-EEG, sensory and event-related potentials. PLoS ONE 8:e57069. 10.1371/journal.pone.0057069 23460826 PMC3584112

[B19] Rohaut B. Faugeras F. Chausson N. King J. R. Karoui I. E. Cohen L.. (2015). Probing ERP correlates of verbal semantic processing in patients with impaired consciousness. Neuropsychologia 66, 279–292. 10.1016/j.neuropsychologia.2014.10.014 25447058

[B20] Rosanova M. Gosseries O. Casarotto S. Boly M. Casali A. G. Bruno M. A.. (2012). Recovery of cortical effective connectivity and recovery of consciousness in vegetative patients. Brain 135 (Pt 4), 1308–1320. 10.1093/brain/awr340 22226806 PMC3326248

[B21] Thut G. Veniero D. Romei V. Miniussi C. Schyns P. Gross J. (2011). Rhythmic TMS causes local entrainment of natural oscillatory signatures. Curr. Biol. 21, 1176–1185. 10.1016/j.cub.2011.05.049 21723129 PMC3176892

